# The adenosine generating enzymes CD39/CD73 control microglial processes ramification in the mouse brain

**DOI:** 10.1371/journal.pone.0175012

**Published:** 2017-04-04

**Authors:** Marina Matyash, Oleksandr Zabiegalov, Stefan Wendt, Vitali Matyash, Helmut Kettenmann

**Affiliations:** 1 Cellular Neurosciences, Max-Delbrück Center for Molecular Medicine in the Helmholtz Association, Berlin, Germany; 2 Department of Neuropathology, Charité-Universitätsmedizin Berlin, Berlin, Germany; Indiana University School of Medicine, UNITED STATES

## Abstract

Microglial cells invade the brain as amoeboid precursors and acquire a highly ramified morphology in the postnatal brain. Microglia express all essential purinergic elements such as receptors, nucleoside transporters and ecto-enzymes, including CD39 (NTPDase1) and CD73 (5'-nucleotidase), which sequentially degrade extracellular ATP to adenosine. Here, we show that constitutive deletion of CD39 and CD73 or both caused an inhibition of the microglia ramified phenotype in the brain with a reduction in the length of processes, branching frequency and number of intersections with Sholl spheres. *In vitro*, unlike wild-type microglia, cd39^-/-^ and cd73^-/-^ microglial cells were less complex and did not respond to ATP with the transformation into a more ramified phenotype. In acute brain slices, wild-type microglia retracted approximately 50% of their processes within 15 min after slicing of the brain, and this phenomenon was augmented in cd39^-/-^ mice; moreover, the elongation of microglial processes towards the source of ATP or towards a laser lesion was observed only in wild-type but not in cd39^-/-^ microglia. An elevation of extracellular adenosine 1) by the inhibition of adenosine transport with dipyridamole, 2) by application of exogenous adenosine or 3) by degradation of endogenous ATP/ADP with apyrase enhanced spontaneous and ATP-induced ramification of cd39^-/-^ microglia in acute brain slices and facilitated the transformation of cd39^-/-^ and cd73^-/-^ microglia into a ramified process-bearing phenotype *in vitro*. These data indicate that under normal physiological conditions, CD39 and CD73 nucleotidases together with equilibrative nucleoside transporter 1 (ENT1) control the fate of extracellular adenosine and thereby the ramification of microglial processes.

## Introduction

Microglia mediate innate immune responses in the brain and, as recently became evident, are involved in tissue remodeling during development as well as in synaptic plasticity in the adult brain [1, 2]. Microglial cells enter the brain early in development as amoeboid cells and transform postnatally into a ramified phenotype characterized by a small soma and extensively branched processes [3, 4]. Highly ramified microglia is a sign of a healthy brain [2].

A characteristic feature of these cells is a specific form of cell motility—they continuously extend and retract their processes and thereby scan the brain environment while their soma remains in a fixed position [5, 6]. In response to a focal injury, microglia change its motility mode by moving the processes toward the injury site [5, 7]. This microglial response plays a protective role as its inhibition leads to increased areas of tissue damage [8]. Moreover, microglia contact synapses and can thereby control their functional state [9, 10]. Both motility and phagocytosis of microglia are tightly regulated by purinergic signaling. The targeted extension of the microglia process is controlled by ATP and is mediated by activation of the P2Y12 subtype of purinergic receptors [7, 11]. Activation of P2Y12 receptors and A3 adenosine receptors is required for chemotaxis [7, 12, 13], whereas activation of P2Y6 receptors enhances phagocytosis [14]. On the other hand, activation of A2A adenosine receptors mediates microglial process retraction during inflammation [15].

Purinergic signaling is mediated by extracellular purine and pyrimidine ligands and exerts a variety of biological effects. The purinergic elements consist of ecto-nucleotide-metabolizing enzymes hydrolyzing nucleoside phosphates, purinergic receptors classified as P1 for adenosine and P2 for nucleoside tri-/diphosphates, nucleoside transporters, and finally, adenosine deaminase, which converts adenosine to inactive inosine. These purinergic elements have tightly concerted actions under physiological conditions and trigger, maintain and terminate purinergic signaling [16–24].

Extracellular nucleotide metabolism is mediated by a cascade of membrane-bound nucleotidases [22, 23, 25]. The currently known nucleotidases include the ecto-nucleotidase triphosphate diphosphohydrolase (NTPDase) family, ecto-pyrophosphatase/phosphodiesterase (NPP) family, ecto-5’-nucleotidase /CD73 and ecto-alkaline phosphatase. Among the NTPDase family, only NTPDase1, also termed CD39, hydrolyses ATP and ADP with similar rates, whereas other family members have a preference for ATP. CD39 regulates cell chemotaxis and, in the brain, it is exclusively expressed by microglia [26–28].

Adenosine is the final bioactive product of ATP breakdown. A critical role of local adenosine generation in the ATP-induced responses has long been recognized. For example, inhibition by extracellular ATP of hippocampal synaptic transmission requires ATP hydrolysis to adenosine. [29, 30]. Moreover, alterations in CD39 expression correlate with a reduction in adenosine signaling [31, 32], and ATP/ADP breakdown is also required for ATP-induced chemotaxis since adenosine may help establish a gradient for directed cell extension [13, 33]. The molecular mechanism for microglial process ramification in the brain is not well understood. Here, we focused on the role of extracellular adenosine in the regulation of microglial morphology under normal, non-pathological conditions. We demonstrated that the CD39/CD73 enzymatic pathway and adenosine transport via ENT1 control formation, ramification and spatial distribution of microglia processes through regulation of extracellular adenosine.

## Materials and methods

### Ethics statement

All procedures involving handling of living animals were performed in strict accordance with the German Animal Protection Law and were approved by the Regional Office for Health and Social Services in Berlin (Landesamt für Gesundheit und Soziales, Berlin, Germany, Permit Number X9023/12). Adult mice were sacrificed by cervical dislocation or intraperitoneal injection of Narcorene^®^, which is a commercial animal euthanasia injectable solution. Narcorene^®^ was purchased from Merial GmbH (Hallbergmoos, Germany) and was used strictly according to the manufacturer’s instruction. Active substance in Narcorene^®^ is pentobarbital sodium salt (5-Ethyl-5-(1-methylbutyl)-2,4,6(1*H*,3*H*,5*H*)-pyrimidinetrione sodium salt) (16 gram per 100 mL of injection solution). Newborn mouse pups were euthanized by decapitation. All efforts were made to minimize suffering.

### Animals

Mice were on C57BL/6J genetic background. cd39^-/-^ and cd73^-/-^ knockout mice were described previously [32, 34]. Double cd39^-/-^/cd73^-/-^ knockout mice were generated after crossing of heterozygotes cd39^-/+^/cd73^-/+^, that were obtained after crossing of cd39^-/-^ with cd73^-/-^ mice, respectively. Double cd39^-/-^/cd73^-/-^ knockout mice were born in time and developed normally. MacGreen is a reporter mouse line [35] expressing enhanced green fluorescent protein (EGFP) under colony stimulating factor 1 receptor (CSF1R) promoter; in brains of MacGreen mice only microglia express EGFP. Subsequently, MacGreen and cd39^-/-^mouse lines were crossed, MacGreen/cd39^+/+^ (MacGreen/wild-type) and MacGreen/cd39^-/-^ mice were used to visualize living microglia in acute brain slices by the mean of two-photon confocal microscopy. Mice of either sex were used for experiments. Mice were kept under a 12 hour/12 hour dark-light cycle with food and water supply *ad libitum*. All experiments were performed according to the guidelines of the German law for animal welfare.

### Reagents

ATP, ADP, AMP, adenosine, dipyridamole, apyrase and dimethylsulfoxid (DMSO) were purchased from Sigma (Taufkirchen, Germany); 2-methylthioadenosine diphosphate trisodium salt (2meSADP) was from Tocris (Bristol, UK); DMEM, Dulbecco’s Phosphate-Buffered saline without Ca^2+^ and Mg^2+^, trypsin/EDTA solution, HEPES was obtained from Thermo Scientific (Walldorf, Germany); NaCl, KCl, MgCl_2_, CaCl_2_, K_2_HPO_4_, NaHCO_3_, D-glucose, paraformaldehyde (PFA), Triton X-100, Tween 20, bovine serum albumin (BSA), and sucrose were from Roth (Karlsruhe, Germany). Heparin (Heparin-Natrium-250 000-ratiopharm) was from Ratiopharm (Ulm, Germany), Narcorene^®^ from Merial GmbH (Hallbergmoos, Germany), ticagrelor was purchased from Cayman Chemical (Hamburg, Germany); concentrated stock solutions of ticagrelor and dipyridamole were prepared in DMSO and diluted in artificial cerebrospinal fluid (ACSF) or cell culture medium right before use.

### Preparation of acute brain slices

Animals were killed by cervical dislocation, brains were dissected, mounted onto the stage of a vibrating-blade rotary microtome (Microm HM 650V from Thermo Scientific) and sliced in an ice-cold artificial cerebrospinal fluid (ACSF) containing (mM): NaCl 134; KCl 2.5; MgCl_2_ 1.3; CaCl_2_ 2; K_2_HPO_4_ 1.25; NaHCO_3_ 26; D-glucose 10; pH 7.4, osmolality ~ 300 mOsm. The ACSF was saturated with carbogen [36]; 300 μm-thick coronal brain slices containing the somatosensory cortex were prepared.

Thereafter slices were transferred into an ACSF at room temperature, which was permanently gassed with carbogen. Acute brain slices were used either for imaging of living microglia by the mean of two-photon microscopy or fixed in a solution of 4% paraformaldehyde (PFA), and used for 3-dimensional morphological analysis of microglia.

### Preparation of frozen sections and immunocytochemistry

Mice were euthanized with intraperitoneal (i.p.) injection of Narcorene^®^ (2.5 mL/kg), perfused transcardially with 10 mL of 0.9% NaCl solution containing 1 U/mL of heparin to clear the intravascular compartment from blood cells followed by 50 mL of 4% paraformaldehyde in 0.1 M phosphate buffer, pH 7.4 (4% PFA). The brains were dissected, fixed in 4% PFA for 12 h at 4°C, cryoprotected by sequential incubation for 24 h at 4°C in 20% and 30% sucrose solution in 0.1 M phosphate buffer, pH 7.4, then frozen by immersion for 30 seconds into 2-methylbutane cooled by dry ice, and stored at -80°C. Brains were sectioned coronal into 70 μm-thick slices using a sliding microtome (Leica SM2000R, Nussloch, Germany).

Primary rabbit polyclonal antibody against mouse ionized calcium binding adaptor molecule 1 (iba-1) (Wako Pure Chemicals Industries, Osaka, Japan) and secondary donkey polyclonal Alexa fluor-488 labelled antibody (Thermo Scientific) were used to label microglia. Immunohistochemistry was performed on free-floating sections under continuous gentle agitation. First, sections were transferred into 24 well-plate, washed (3 times, 5 minutes each) with TBS (20 mM TRIS, 136 mM NaCl, pH 7.4), then incubated in a detergent-containing blocking solution (1% Triton X-100, 5% BSA, 5% donkey serum in TBS) for 1 hour at room temperature, followed by a sequential incubation for 24 h at 4°C in a detergent-free blocking solution containing either primary or secondary antibody. After the incubation with the antibody, sections were washed (3 times, 10 min each) at room temperature in TBS containing 1% BSA and 0.1% Tween 20. Finally, sections were immersed into a DAPI (300 nM) solution in TBS, mounted onto glass slides (Superfrost^®^Plus), embedded into Vectashield (Biozol, Eching, Germany), and kept at 4°C.

### Confocal microscopy and 3-dimensional quantitative morphology of microglial processes

High-resolution 3-dimensional (3D) panoramic images (X x Y x Z dimension in μm: 1200 x 1200 x 50) of somatosensory cortex (Layer 1–6) were obtained using the tile-scan function of an LSM710 confocal microscope (Zeiss, Oberkochen, Germany) equipped with motorized table and controlled by Zen2010 software. Z-stacks with 1.0 μm steps were acquired using a 40x oil immersion objective (Plan-Apochromat) with a numeric aperture of 1.3 and working distance of 0.19 mm, and the pinhole size was set to 1 Airy unit. Morphological analysis of microglia was performed on 3-dimensional fluorescence images using Imaris 7.6.4 algorithms without conversion of the images into 2-dimensional maximum intensity projections. Microglial cells in which the nucleus was at least 15 μm away from the image border were selected for analysis. The following parameters were quantified: cumulative length of the processes, number of branch and terminal points, number of processes intersecting Sholl spheres (Sholl intersections), length and number of process segments (a segment is a part of a process which locates either between the soma and nearest branch point, two sequential branch points, or a sequential branch and terminal point). The branch level of each segment was determined as follows: the initial segment level is 1; at each branching point the process segment with a smaller diameter sequentially increases segment branch level, while the segment with a greater diameter maintains the same branch level; if the segments were of similar size, they were both assigned a higher segment level (Imaris 7.6.4, manufacture instructions). In wild-type microglial cells, processes of two cells sometimes intermingled. Although tracing was performed automatically by the algorithm, we individually verified that processes originated from one defined cell. False connections were removed manually which were commonly less than 1%. The number of Sholl intersections was defined as the number of process intersecting concentric spheres, defining the spatial distribution of segments as a function of distance from the soma (Sholl analysis). All spheres have their center at the soma (beginning point) with a 5 μm step resolution for the spheres. The coordinates of process terminals (terminal points) and cell somata were determined, and the number of terminal points located in one of four different quadrants (dorsal_right_, dorsal_left_, ventral_right_, ventral_left_) or in one of two locations (between the slice surface and cell soma, between the cell soma and depth of slice) with respect to the position of the cell soma in the cortex was quantified for every individual microglia. In acute brain slices, at least 20 individual microglial cells per experimental condition were analyzed. The volume of membranous enlargements that appeared along the processes in acute brain slices was automatically quantified on 3-dimensional fluorescence images using the Imaris 7.6.4 algorithm, and at least 3 random images (X x Y x Z dimension in μm: 210 x 210 x 30) per one brain slice were analyzed. An absolute number of iba-1 labelled microglial cells were automatically quantified in volumes with a fixed size (X x Y x Z dimension in μm: 210 x 210 x 50); at least 10 random volumes per one brain slice and at least 3 brain slices per animal were analyzed. Microglia density was normalized to 1 mm^3^ of brain tissue.

### Two-photon imaging and laser lesion

For two-photon imaging, 300 μm coronal cortical brain slices were obtained either from MacGreen/cd39^+/+^ or MacGreen/cd39^-/-^ mice. EGFP was excited by a Chameleon Ultra II laser (Coherent, Dieburg, Germany) set to a wavelength of 950 nm. Imaging was performed with a two-photon laser scanning microscope (Till Photonics, Gräfelfing, Germany) equipped with a water-immersion objective (40x, NA 0.8, Olympus, Hamburg, Germany). We imaged a 60-μm thick z-stack with a step size of 3 μm covering a field of 307 x 307 μm.

Laser lesions were created in brain slices by focusing the laser beam, set to a wavelength of 730 nm and maximal power, at 40 μm depth. This procedure resulted in lesions approximately 19 ± 6 μm in size (between 10 and 34 μm, measured as the longest dimension of the lesion) in the middle of the observed region. Records were obtained from acute brain slices which were kept in ACSF for at least 1 hour (but not longer than 3 h) after the preparation. Image J software (http://imagej.nih.gov/ij/) was used for data analysis as described elsewhere [5, 37]. Briefly, the sequences of 3-dimensional images were converted into sequences of 2-dimensional images by the maximum intensity projection algorithm. Microglial response to the focal lesion was quantified as a shift of EGFP fluorescence from distal into proximal circular regions surrounding the lesion site; the microglial response is therefore given as R(t) = (Rx(t)—Rx(0)/Ry(0).

### Endpoint laser lesion assay in brain slice

Endpoint laser lesion assay was performed as following: brain slices from three MacGreen/wild-type and five MacGreen/cd39^-/-^ mice were prepared and incubated in a perfusion chamber. A focal laser lesion was applied as described above and subsequently slices were maintained for 15 minutes at room temperature either in ACSF alone (ACSF) or in ACSF supplemented with adenosine (1 μM) and dipyridamole (20 μM). Subsequently slices were fixed with 4% PFA, washed in TBS (3 times, 5 minutes each), mounted onto glass slides and embedded in Vectashield medium. High-resolution 3-dimensional (3D) fluorescence images were acquired from the brain slices containing a laser lesion spot. The spot could be located based on it intense auto-fluorescence. We acquired 30-μm thick Z-stacks with a step size of 0.5 μm covering a field of 137 x 137 μm using LSM710 confocal microscope as described above. 9 and 32 laser lesions were set in brain slices derived from three MacGreen/wild-type and five MacGreen/cd39^-/-^ mice, respectively. In MacGreen/cd39^-/-^ mice microglial responses to 17 (ACSF alone) and 15 (ACSF with adenosine and dipyridamole) laser lesion were analyzed. Image stacks were processed using Fiji distribution of ImageJ software (https://fiji.sc/). Absolute number of fluorescent objects corresponded to microglial processes and cell somata were counted using 3D Object Counter plug-in. Microglial somata with the volume larger than 120 μm^3^ as well as small objects with the volume smaller than 1.0 μm^3^ were excluded from data analysis.

### Cultivation and morphological analysis of primary neonatal microglia

1-Day-old mouse pups were killed by decapitation. For cultures of cd39^-/-^ and cd73^-/-^ microglia individual newborn animals were tagged and genotyped on the day of birth (P0). Only verified knockout pups were used one day later (P1) for preparing cell cultures. Primary cultured neonatal microglia were prepared as described elsewhere [38]; microglia were collected from mixed cultures, seeded into 35 mm Glass Bottom Dishes with 10 mm glass insert (MatTek Corporation, Ashland, USA) at a cell density of 10^4^ cells per 1 cm^2^, and further cultivated in 2 mL of serum-free DMEM medium supplemented with 0.5 mM glutamine. Imaging of living microglia (time-lapse series or random images) was conducted in the Nikon BioStation IM (Nikon GmbH, Duesseldorf, Germany) equipped with a phase-contrast microscope. Cell culture medium was regularly changed (2 mL) every 2^nd^ day. At the given cell density, approximately 30% of the glass surface was covered with microglia; therefore, it was possible to measure the area and the perimeter of individual cells as well as the changes in these parameters over time. As indicated in the results, microglia were cultivated in a serum-free medium alone or in the presence of ATP, ADP, AMP, adenosine, ticagrelor (5 μM) or dipyridamole (20 μM). To assure non-biased manual morphometric analysis, phase-contrast images were blindly processed. Cell area and perimeter were measured using standard tools of Fiji software by manually tracing cell profiles. We selected for cells which were individually recognized. Cell circularity was quantified according to the following equation: circularity = 4pi*(cell area/cell perimeter^2^). Data show circularities of at least 200 cells per one experimental condition out of 3 independent experiments. The cell area that was explored by microglial processes were measured in a time-lapse series of living cells at 15 min, 30 min and 60 min using a Fiji algorithm. The time-series were converted into a Z-projection, and the final minimal intensity image represented an overlay of all sequentially acquired images; the explored area was quantified as a difference between the areas measured on the final minimal intensity image and on the first image in the time-series.

## Results

### Constitutive genetic deletion of CD39, CD73 or both enzymes attenuates microglial process ramification

**Fig 1:**
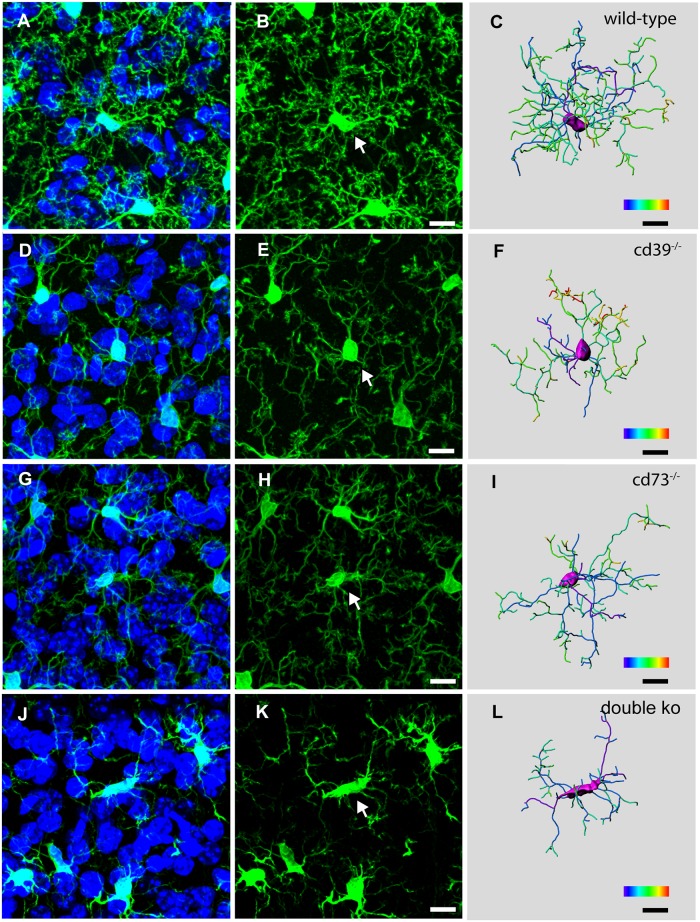
Constitutive deletion of CD39 and/or CD73 attenuates microglial process ramification. Three-dimensional confocal fluorescence images show iba-1/Alexa fluo-488 labelled microglia (left and middle panels) and DAPI-labelled nuclei (left panels), and 3-dimensional reconstruction of an individual microglial cell (right panel) in the somatosensory cortex of wild-type (A—C), cd39^-/-^ (D—F), cd73^-/-^ (G—I) and cd39^-/-^/cd73^-/-^ (double ko) (J—L) adult (P56) mice are shown; white arrows mark a single microglial cell of which the 3-dimensional reconstructed image is shown in the right panel. Color bar scale decodes the branch level (from 1 to 7) of individual microglial segments. Scale bar denotes 10 μm.

**Fig 2:**
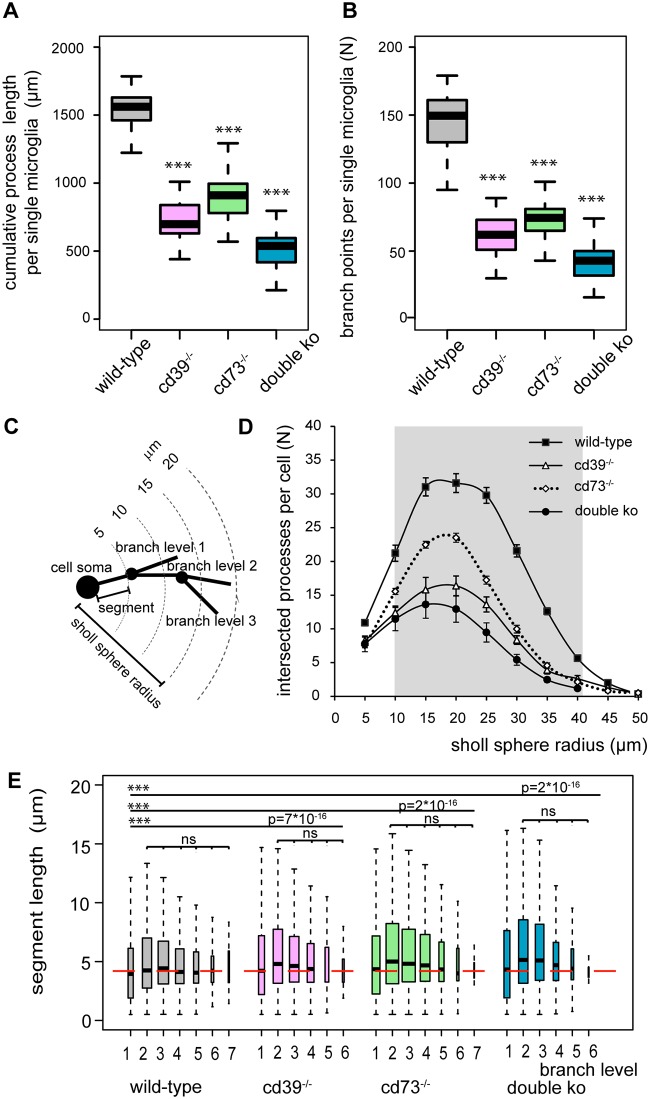
Quantification of 3-dimensional microglial process ramification shows that microglia from adult (P56) cd39^-/-^, cd73^-/-^, and double knockout mice have a less complex morphology in the somatosensory cortex compared to those from wild-type mice. Individual microglial cells from wild-type (grey, N = 46), cd39^-/-^ (pink, N = 46), cd73^-/-^ (green, N = 70) and double knockout (cd39^-/-^/cd73^-/-^) (blue, N = 128) mice were analyzed using Imaris 6.7.4; N represents the number of individual cells. Box plots show that cumulative processes length (A) was shorter with a fewer number of branch points (B) in the knockout microglia. C is a scheme to illustrate the Sholl analysis. The number of processes intersecting concentric rings every 5 μm is shown in D. The grey area marks the Sholl sphere radius where a significant (p < 0.001) reduction in the number of Sholl intersections in knockout microglia in comparison to wild-type microglia was found. (E) Box plots show the length of individual segments for each branch level (1 to 6) of wild-type (grey, N = 13650), cd39^-/-^ (pink, N = 5709), cd73^-/-^ (green, N = 10740) and double knockout (blue, N = 11778) microglial cells. In each box plot, the bar demarks the median, the box demarks the range from 25% to 75% of the data, and the dotted lines indicate the entire range of data. N represents the number of analyzed segments. The depth of process branching (branch level) was reduced whereas the length of individual segments was increased in knockout mice; the red dashed line shows the median of the segment length in wild-type microglia. ^ns^ denotes p > 0.5 and *** denotes p < 0.001.

To perform a quantitative 3-dimensional morphological analysis and to compare the complexity of microglia process organization in the brains of wild-type and knockout mice, we used immunolabeling of iba-1 [39], a specific microglia cell marker, to visualize microglial morphology in frozen sections of PFA-perfused brains. Fig 1 shows representative examples of microglial cells in cortices of adult (postnatal day 56, P56) wild-type, cd39^-/-^, cd73^-/-^ and double knockout (cd39^-/-^/cd73^-/-^) mice. Microglial morphology in knockout animals was less complex, and we analyzed the morphological features with a quantitative approach. The cumulative length of microglial processes in wild-type mice was 1500 ± 200 μm, but it was significantly reduced to 713 ± 147 μm and 904 ± 154 μm in cd39^-/-^ and cd73^-/-^ mice, respectively (Fig 2A). While processes of wild-type microglia underwent extensive branching and had 150 ± 29 branch points per cell, this parameter was significantly reduced to 61 ± 15 and 74 ± 15 in cd39^-/-^ and cd73^-/-^ mice, respectively (Fig 2B). In double knockout mice, the cumulative process length and the number of branch points were even further reduced to 537 ± 178 μm and 41 ± 13, respectively, and were significantly (p<0.001) lower than those of microglia from the brains of cd39^-/-^ and cd73^-/-^ knockout mice (Fig 2A). Furthermore, 3-dimensional Sholl analysis demonstrated that microglia can extend processes up to 50 μm away from the cell soma, but 80% of all microglial processes were located within a 40 μm radius around the soma (Fig 2C and 2D). The number of microglial processes intersecting the Sholl spheres within the radius from 10 to 40 μm were significantly reduced in the brains of cd39^-/-^, cd73^-/-^ and double knockout mice (Fig 2D). Every primary microglial process that originated at the cell soma formed multiple branches, which branched further. We analyzed these process segments which were defined as portions of the process located either between the soma and a first branch point, between two sequential branch points, or between the last branch point and a terminal point. After a branch level (see materials and methods) was assigned to every individual segment, we found that processes branched regularly and had up to 7 branch levels, and the length of the segment was un-correlated with segment branch level (Fig 2E). Wild-type microglia had a higher branching frequency and significantly shorter individual segments compared to microglia in cd39^-/-^, cd73^-/-^ and double knockout mice; medians of the segment length were 4.2 ± 3.7 μm, 4.6 ± 4.2 μm, 4.7 ± 4.3 μm and 4.9 ± 4.3 μm, respectively (Fig 2E).

Next, the orientation of microglial processes was analyzed by the spatial distribution of the process terminals (terminal points) within the four quadrants around the microglia soma. While each quadrant contained an average of 25 ± 10% of the cumulative number of terminal points in the wild-type microglia (N = 46, N represents the number of analyzed microglial cells), individual microglial cells in the cd39^-/-^ (N = 46), cd73^-/-^ (N = 70) and double knockout (N = 128) mice were less symmetric (S1 Fig). Cells were classified as symmetric if the number of terminal points in each of the four quadrants around the microglia soma was between 15 to 35%. In wild-type mice, 47% of individual microglia were symmetric; in cd39^-/-^, cd73^-/-^ and double knockout mice, a significantly (p<0.01) smaller number of individual cells, namely 20%, 30% and 23% of microglia, respectively, showed a symmetrical distribution of process terminals around the cell soma (S1 Fig). These results indicate that the activity of CD39 and CD73 is critical for the formation, branching and spatial distribution of microglia processes *in vivo*.

### Deletion of CD39 and CD73 results in decreased microglial density in the brain

Similar to the distribution in wild-type mice, microglia in adult (P56) knockout mice were evenly distributed across all layers of the cortex. The microglia density in wild-type mice was 14465 ± 2307 cells/mm^3^ in the somatosensory cortex (n = 11, n refers to the number of individual animals per group), but it was significantly reduced (p<0.01) by approximately 20% in cd39^-/-^ and cd73^-/-^ mice as well as in double knockout mice: mean values were 11441 ± 1633 (n = 11), 11730 ± 1649 (n = 4), and 12807 ± 1497 (n = 4), respectively.

### Brain slicing induces rapid degradation and retraction of microglia processes

**Fig 3:**
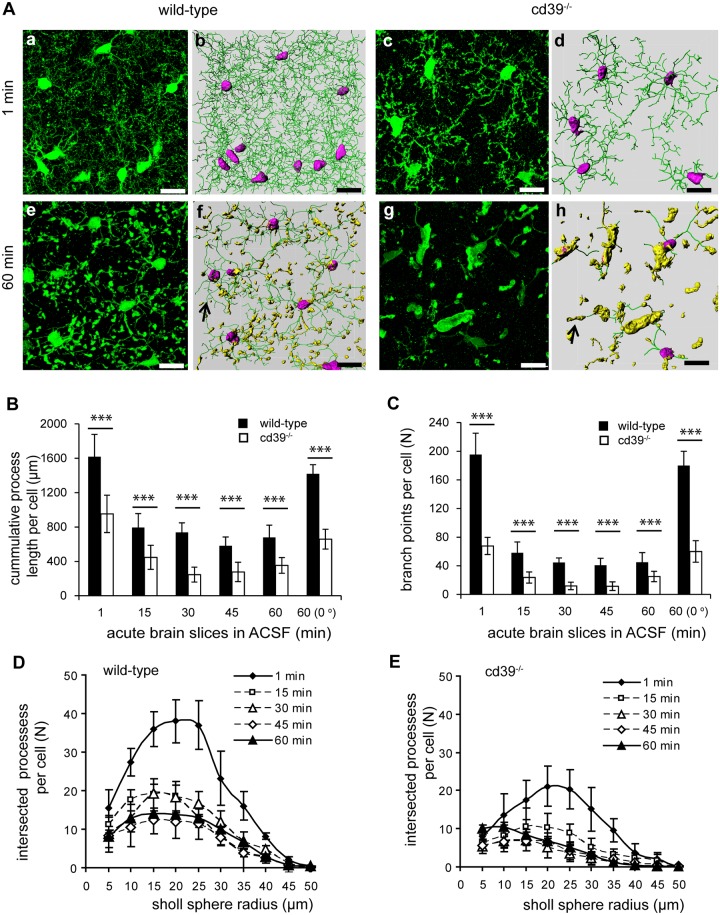
Quantification of 3-dimensional microglial process ramification shows a rapid reduction in the ramified phenotype of microglia in acute brain slices of adult (P56) wild-type and cd39^-/-^ mice. (A) Three-dimensional fluorescence confocal images (a, c, e, g) and the corresponding rendered images of processes (b, d, f, h) of microglia in acute slices of somatosensory cortex of wild-type (left) and cd39^-/-^ (right) mice are shown; the green lines (marked by arrow) on rendered images show the processes; the microglial cell soma is labeled in pink; and membranous swellings along the processes are in yellow. (B) Cumulative length of microglial processes and (C) the number of branch points were quantified 1 minute and then every 15 min after slicing of brains of wild-type (black bars) and cd39^-/-^ (white bars) mice. The last column shows the values from slices maintained in ACSF for 60 min at 0°C. (D) The number of Sholl intersections per microglia were measured (as described in the legend to Fig 2) at different time points after slicing brains of wild-type (left) and cd39^-/-^ (right) mice. Scale bar denotes 15 μm. ^ns^ denotes p > 0.5 and *** denotes p < 0.001.

In the brain, the expression of CD39 is restricted to microglia and endothelial cells, while the expression of CD73 is not that selective [26]; moreover, the breakdown of extracellular ATP to adenosine is initiated by CD39 [22]. Therefore, we focused on cd39^-/-^ mice to test the impact of extracellular ATP/ADP degradation on microglia ramification in the model of acute cortical brain slices. Dynamic changes in the organization of microglial processes were analyzed at defined times after preparing acute brain slices. One minute after brain slicing, similar differences were found with respect to the degree of microglia ramification between wild-type and cd39^-/-^ mice as observed in frozen sections of PFA-perfused brains (Fig 3A). The cumulative length of processes per cell was significantly shorter (58%), with fewer Sholl intersections and branch points (35%), in microglia from cd39^-/-^ mice compared to those from wild-type mice (Fig 3B–3D). At 15 min after slicing, we found that in wild-type microglia, the cumulative length of microglia processes (from 1600 ± 300 μm to 825 ± 175 μm) and the number of branch points (from 195 ± 30 to 60 ± 15) was reduced (Fig 3B and 3C). In acute brain slices isolated from cd39^-/-^ mice, microglia started out with shorter processes and fewer branch points, but these values decreased further during the maintenance of the slices in ACSF at room temperature (Fig 3B and 3C). The number of process segments per microglia decreased from 403 ± 61 (1 min) after slicing to 123 ± 32 (15 min) in wild-type mice and from 140 ± 23 (1 min) to 50 ± 15 (15 min) in cd39^-/-^ mice. Additionally, the Sholl analysis indicated that a reduction in the number of Sholl intersections occurs within the first 15 min in wild-type and cd39^-/-^ mice (Fig 3D). The structure of the remaining microglial processes also underwent morphological changes. Numerous membranous swellings formed along the processes as well as at their tips (Fig 3A). Immediately after slicing, some segments became larger than the others and appeared as intense fluorescence enlargements with a mean volume of 1.04 μm^3^ and 1.58 μm^3^ in wild-type and cd39^-/-^ brain slices, respectively. The larger structures were often observed at branching sites of the processes. When slices were incubated for 60 min in ACSF at room temperature, the mean volume of membranous enlargements (nuclei-free) increased to 13.35 μm^3^ in wild-type mice and to a significantly (p<0.001) larger volume of 24 μm^3^ in the acute brain slices from cd39^-/-^ mice (see Fig 3Ae, 3Af, 3Ag and 3Ah). This indicates that the transformation of cellular membranes into thin processes was strongly inhibited in the acute brain slices of cd39^-/-^ mice, which led to a collapse of approximately 90% of the ramified processes when compared to wild-type microglia.

### Elongation of microglia processes in acute brain slices requires ATP and its catabolic product adenosine

**Fig 4:**
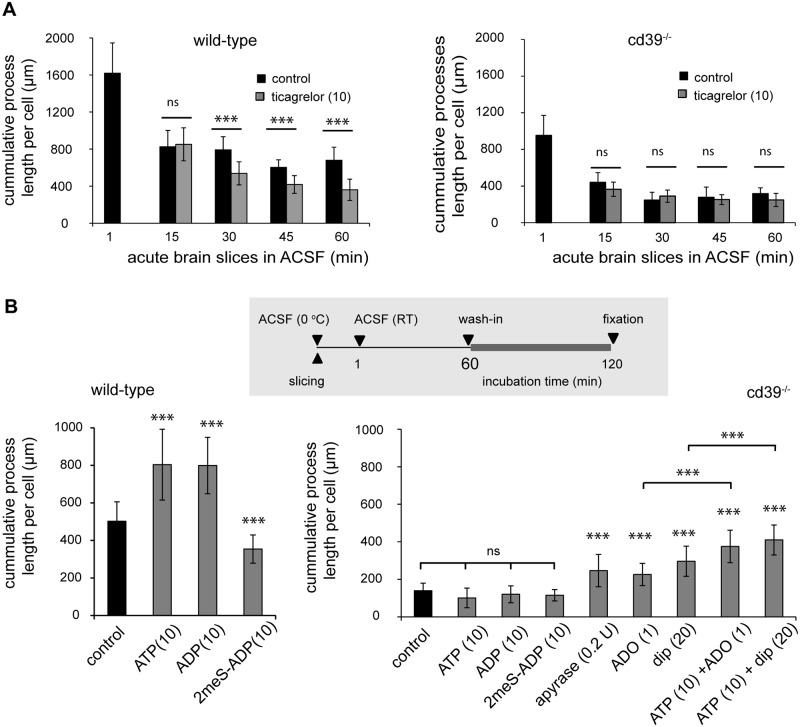
In acute brain slices, microglial process growth is promoted by constitutive activity of P2Y12 receptors and availability of extracellular adenosine. (A) As described in the legend to Fig 3, the cumulative process length of microglia was measured 1 minute and then every 15 min after acute slice preparation. In comparison to control (black bars) conditions, constitutive inhibition of P2Y12 receptors with ticagrelor (grey bars, 10 μM) significantly decreased the cumulative length of microglial processes in acute brain slices from wild-type (left) but not from cd39^-/-^ mice (right). (B) On top, the scheme indicates the experimental arrangement. In wild-type microglia (left) but not in cd39^-/-^ mice (right), ATP (10 μM) and ADP (10 μM) increased the cumulative length of microglial processes in comparison to control (black bars) slices. 2meS-ADP (10 μM) even decreased process length in acute brain slices of wild-type but not of cd39^-/-^ mice. Application of apyrase (0.2U), adenosine (ADO, 1 μM), dipyridamole (dip, 20 μM), and a combined application of ATP and adenosine or ATP and dipyridamole increased the cumulative length of microglial processes in cd39^-/-^ mice in comparison to control (black bars) conditions. ^ns^ denotes p > 0.5 and *** denotes p < 0.001.

P2Y12 receptors are involved into the elongation of microglia processes in response to ATP [7, 11]. *In vivo*, microglia constantly extend and retract their processes [6], and thus, the final length and, probably, branching of microglia processes depend on the balance between signals supporting extension and signals mediating retraction. Continuous incubation of brain slices in the presence of the P2Y12 inhibitor ticagrelor (10 μM) led to a gradual reduction in microglia cumulative process length and the number of branches (not shown) in acute brain slices of wild-type but not of cd39^-/-^ mice when compared to ACSF alone (Fig 4A). Furthermore, low concentrations of ATP (10 μM) and ADP (10 μM) increased but 2meS-ADP (10 μM), a non-hydrolysable analogue of ADP, decreased microglia cumulative processes length and the number of branches (not shown) in acute brain slices of wild-type but not of cd39^-/-^ mice (Fig 4B). This indicates that not only constitutive activity of P2Y12 receptors but also constitutive ATP/ADP breakdown by NTPDase1 (CD39) is required for spontaneous and ATP-induced elongation and ramification of microglia processes.

Next, we tested whether extracellular adenosine is involved in spontaneous and ATP-induced elongation and ramification of microglia processes. Previously we have demonstrated that hydrolysis of exogenously applied ATP and ADP was strongly inhibited in acute brain slices of cd39^-/-^ mice [38]; subsequently, constitutive degradation of endogenous extracellular ATP could also be attenuated and led to a reduction in the concentration of endogenously generated extracellular adenosine. To elevate the concentration of extracellular adenosine, acute brain slices of cd39^-/-^ mice were incubated with adenosine, dipyridamole or apyrase. Dipyridamole inhibits the equilibrative nucleoside transporter 1 (ENT1) thereby elevating the concentration of endogenously generated extracellular adenosine [30]; apyrase hydrolyses extracellular ATP/ADP to AMP which can be further converted to adenosine by CD73 or ecto-alkaline phosphatase. In acute brain slices of cd39^-/-^ mice, adenosine (1 μM), dipyridamole (20 μM), and apyrase (0.2 U) significantly increased the cumulative length of microglial processes (Fig 4B); the number of branch points was also increased by approximately 25%. Moreover, co-application of ATP and adenosine or ATP and dipyridamole led to a further significant increase in the cumulative length of microglial processes compared to adenosine or dipyridamole alone (Fig 4B). These data show that in the absence of CD39, an elevation of extracellular adenosine rescued the effect of ATP on the ramification of microglia processes, indicating that co-stimulation of P2 and P1 adenosine receptors is required for ATP-induced elongation of microglial processes in the acute brain slice.

### Chemotactic response of microglial processes to ATP/ADP requires extracellular adenosine

**Fig 5:**
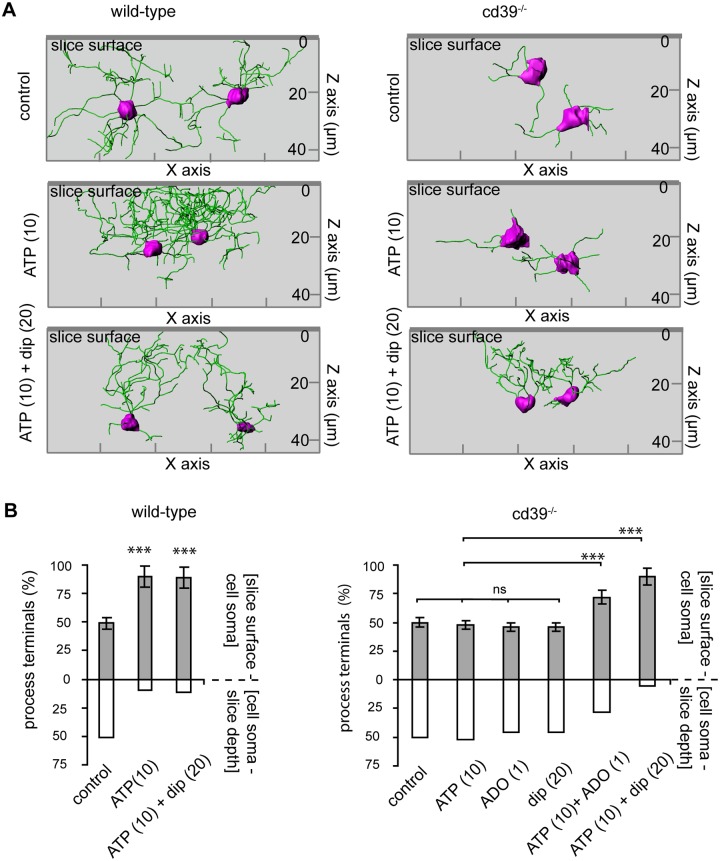
Directional movement of microglial processes towards the surface in acute brain slices requires ATP and adenosine. (A) Representative rendered images of 3-dimensional confocal fluorescence images of iba-1/Alexa fluo-488 labelled microglia in acute brain slices of wild-type (left) and cd39^-/-^ (right) mice are shown. The green lines show the processes, and the cell soma is labeled in pink. In wild-type and cd39^-/-^ mice, the microglial processes are randomly distributed around the cell soma in control conditions. In wild-type mice but not in cd39^-/-^ mice, ATP (10 μM) induced re-orientation and elongation of microglial processes towards the slice surface (left two images). Dipyridamole (dip, 20 μM) restored the ability of cd39^-/-^ microglia to re-orient the processes in an ATP gradient. On the Z axis, the coordinate of the slice surface is zero (0). (B) The bars show a distribution of the cumulative number of process terminal points between two compartments: above (grey) and below (white) the position of the microglia soma with respect to the slice surface. ^ns^ denotes p > 0.5, *** denotes p < 0.001.

We noticed that transient exposure of acute brain slices to 10 μM of either ATP or ADP not only triggered an elongation of microglial processes but also changed process orientation. Using 3-dimensional reconstructions of microglia we analyzed the distribution of process terminals (terminal points) in two compartments around microglia soma: above and below the position of microglia soma with respect to the slice surface. While in control conditions the processes were equally distributed around the microglial soma (Fig 5A and 5B), addition of ATP or ADP (not shown) to the slice supernatant induced a re-orientation of processes towards the slice surface. In wild-type mice, approximately 95% of the process terminals were located between the cell soma and the slice surface when analyzed 60 min after an addition of ATP (Fig 5A and 5B). This observation is in line with those of Dissing-Olesen (2014) [11], where shorter exposure to higher ATP concentrations also triggered an outgrowth of microglia processes towards the slice surface. In acute brain slices of cd39^-/-^ mice, ATP did not trigger any re-orientation of processes towards the slice surface, but reorientation of processes did occur after a combined superfusion of the acute brain slice with ATP (10 μM) and adenosine (1 μM) or ATP (10 μM) and dipyridamole (20 μM): 67 ± 5% or 95 ± 3% of process terminals were located above the cell soma, respectively (Fig 5A and 5B). When applied alone, adenosine (1 μM) and dipyridamole (20 μM) did not trigger re-orientation (Fig 5B) but resulted in a spatially uniform distribution of processes around the cell soma. Thus, the ATP/ADP-hydrolyzing activity of CD39 is required for the orientation and elongation of microglia processes in an ATP (and ADP) gradient.

### Microglial process extension in response to focal lesion is severely affected in acute brain slices derived from cd39^-/-^ mice

**Fig 6:**
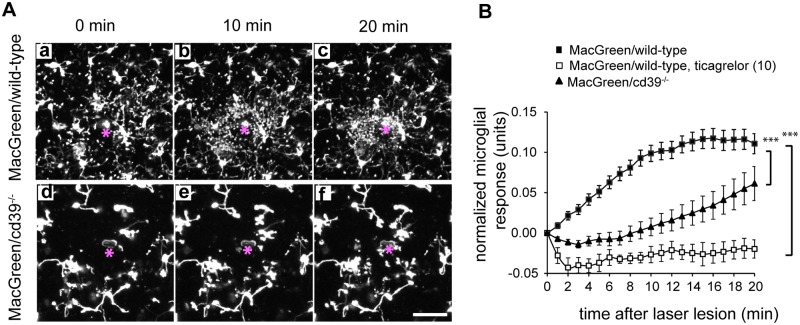
Activity of CD39 is required for directional movement of microglia processes towards a laser lesion in acute brain slices. (A) Movement of microglia processes in acute brain slices of MacGreen/wild-type or MacGreen/cd39^-/-^ mice expressing EGFP under the control of colony stimulating factor 1 receptor promoter (CSF1R) was studied using two-photon time-lapse microscopy. A laser lesion (marked by asterisk) was set, 60 μm-thick Z-stacks with a step size of 3 μm covering a field of 307 x 307 μm were acquired every minute. The extension of the processes toward the laser lesion was strongly attenuated in cd39^-/-^ mice. Images show maximum intensity projections of 60 μm-thick Z-stacks. Scale bar 20 μm. (B). Quantification of laser lesion in MacGreen/wild-type (black squares) and MacGreen/cd39^-/-^ mice (black triangles) over a 20-min time course is shown (n = 8 mice per genotype, 3 slices per an individual mouse), and data are shown as the mean values and standard error of mean. P2Y12 receptor inhibitor ticagrelor (10 μM, white squares) abrogated process movement towards a laser lesion in slices of wild-type mice; slices were pre-incubated with ticagrelor for 20 min before the laser lesion was set. Subsequently, microglial process movement towards the laser lesion was recorded in the presence of ticagrelor. Normalized microglial responses measured 20 min after the laser lesion were compared; significant difference was tested by one-way ANOVA, *** denotes p < 0.001.

Focal laser lesion induced ATP-dependent movement of microglial processes towards the lesion [5]. We therefore compared the microglial response to focal laser lesion in acute brain slices of wild-type and cd39^-/-^ mice by measuring the accumulation of the fluorescently labeled processes within an area around the lesion (for details see materials and methods). In slices from MacGreen/wild-type animals, microglial processes started to extend toward the lesion immediately after the laser injury, and the majority of the processes were accumulated at the lesion site within the first 15 minutes. These microglial processes that moved towards the laser lesion had clearly distinguishable leading edges which were visualized as bright dots (Fig 6A). Their volume ranged from 1.3 to 9.5 μm^3^ (median was 2.5 μm^3^). In acute slices from MacGreen/cd39^-/-^ mice, such response to the laser lesion was temporally delayed (Fig 6B), and the leading edges were not accumulated around the laser lesion. Pre-incubation of brain slices with the P2Y12 inhibitor ticagrelor (10 μM, for 30 min before the laser lesion pulse) completely abrogated the elongation of the processes towards the laser lesion in acute bran slices of MacGreen/wild-type and MacGreen/cd39^-/-^ mice (Fig 6B).

### Addition of adenosine and dipyridamole restores the process extension to a laser lesion in cd39^-/-^ mice

**Fig 7:**
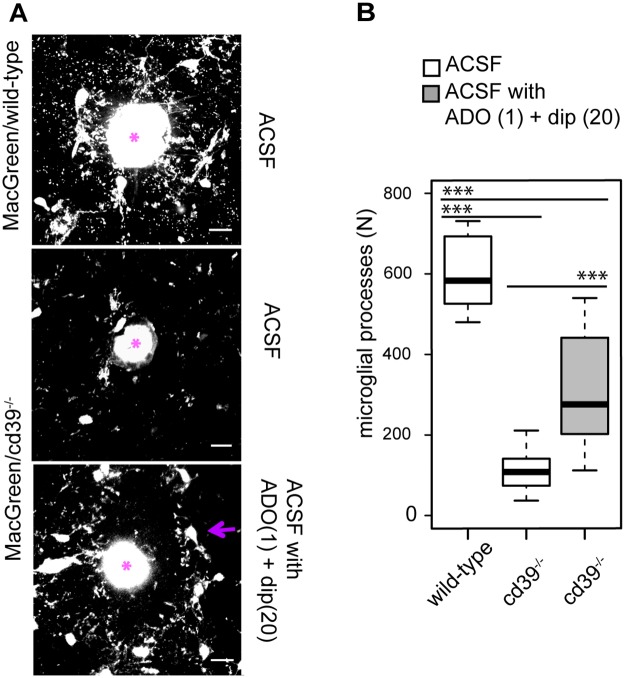
Extracellular adenosine is required for a directional movement of microglial processes towards a focal laser lesion in acute brain slice. (A) Accumulation of microglia processes around a focal laser lesion in an acute brain slice derived from MacGreen/wild-type andMacGreen/cd39^-/-^ mice was studied using confocal microscopy; after a laser lesion was set a brain slice was incubated either with ACSF alone (ACSF) or with ACSF supplemented with adenosine (ADO, 1 μM) and dipyridamole (dip, 20 μM). A laser lesion was set as marked by asterisk. Scale bar 15 μm. (B) Box-plot shows the absolute number of microglial processes that were accumulated around a laser lesion spot in slices from MacGreen/wild-type mice (lesions, n = 9) and in slices from MacGreen/cd39^-/-^ mice in the presence of ACSF alone (lesions, n = 17; white box) and in ACSF supplemented with adenosine (ADO, 1 μM) and dipyridamole (dip, 20 μM) (lesions, n = 15) (grey box); black lines show the median of the data. Significant difference was tested by one-way ANOVA, *** denotes p < 0.001.

Using an endpoint laser lesion assay (see Methods for details) we tested whether a pharmacological blockade of ENT1 by dipyridamole together with an activation of adenosine receptors by adenosine could restore a microglial response to a focal laser lesion in brain slices from MacGreen/cd39^-/-^ mice. In brain slices from MacGreen/wild-type mice multiple microglial processes (583 ± 96) were found around the lesions 15 minutes after the laser pulse. In brain slices from MacGreen/cd39^-/-^ mice significantly lower (p<0.001) number of microglial processes (108 ± 53) accumulated around lesions (Fig 7A and 7B). In comparison to ACSF alone, in the presence of dipyridamole (20 μM) and adenosine (1 μM) we found a significantly increased number of microglial proceses (276 ± 145) around the laser lesions (Fig 7A and 7B). We conclude that a deficiency in the microglial process extension to a focal laser lesion in MacGreen/cd39^-/-^ mice could be partly restored by an elevation of extracellular adenosine indicating that for a rapid extension of microglial processes towards a focal lesion CD39 enzymatic activity is required to provide adenosine in a microglial microenvironment.

### Cultured microglia require P2Y12 receptors, CD39 and CD73 for process motility

**Fig 8:**
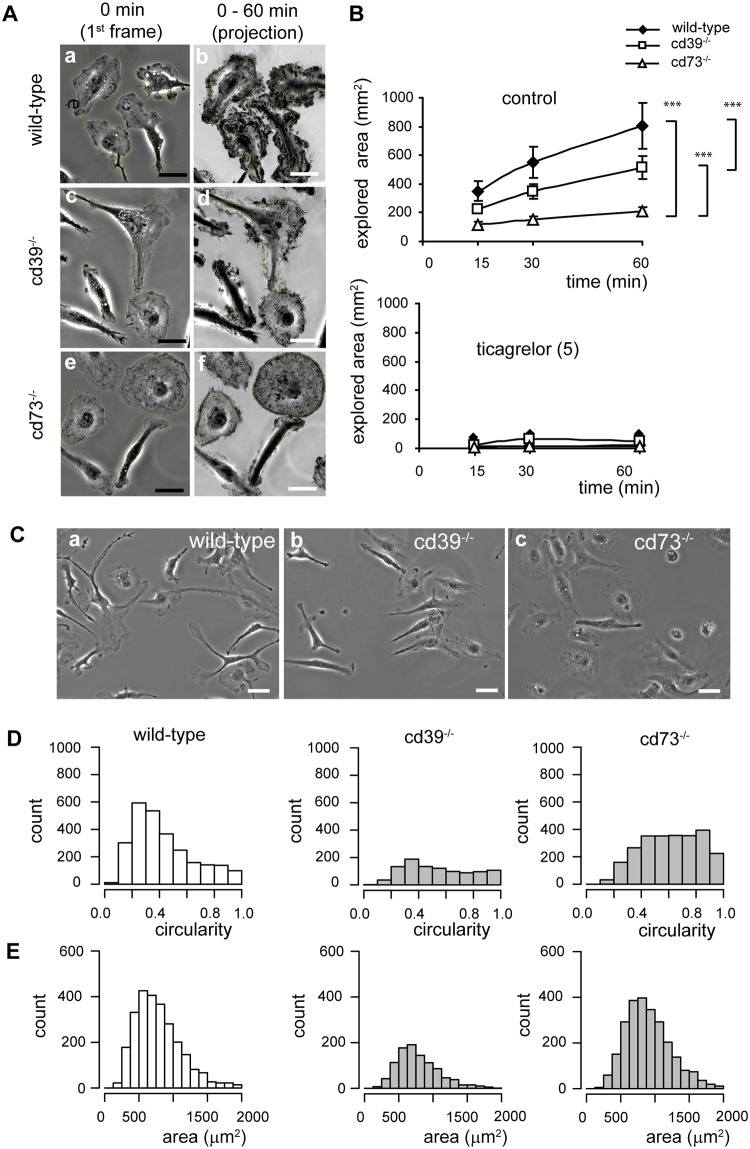
Primary neonatal microglial cells cultured from cd39^-/-^ and cd73^-/-^ mice move more slowly, form fewer protrusions and are more round when compared to wild-type microglia. Cells were monitored by time-lapse video microscopy, and 60 min-long time-series of phase-contrast images were analyzed. (A) A representative first image and a projection of all images in a time-series (60 min) of wild-type (a, b), cd39^-/-^ (c, d) and cd73^-/-^ (e, f) microglia are shown; cells were cultivated under serum-free conditions for no longer than 12 h. Explored areas (during 60 min) were compared, and *** denotes p < 0.001. (B) Summary of the area covered by cells (explored area) during 15, 30 and 60 min. The explored area resulted from protrusions of processes and a translocation of the cell body; explored area was measured as a difference between the entire area which was marked by a cell during the defined period and the cell area measured on the first image. Microglial cells were cultivated under serum-free conditions (control, upper panel) or in the presence of the P2Y12 receptor inhibitor ticagrelor (5 μM, bottom panel). Knockout microglia were significantly slower in comparison to wild-type cells, and ticagrelor abrogated the exploring activity of microglia. (C) Phase-contrast images of wild-type (a), cd39^-/-^ (b) and cd73^-/-^ (c) microglia cultivated for 24 h in a serum-free medium are shown. Scale bar is 20 μm. Histograms show the distribution of cell circularity (D) and cell area (E) of wild-type (N = 2590), cd39^-/-^ (N = 1100) and cd73^-/-^ (N = 2490) microglia; N represents the number of individual cells. For every genotype, circularities and cell areas were collected from at least 1000 cells from 5 independent primary cell cultures; at least 200 individual cells per culture dish were measured. Significant differences were tested by one-way ANOVA, and *** denotes p < 0.001 in comparison to control.

Cultured neonatal microglia constantly move their cell edges and protrusions. To quantify the dynamics of cell edge/protrusion movement, we analyzed time-lapse series of individual cultivated microglial cells and measured the cumulative area (explored area) that was covered by these cells over defined periods of time, namely, within 15, 30 and 60 min (Fig 8A). Within 60 min, each wild-type microglial cell had covered 804 ± 71 μm^2^. The area covered by cd39^-/-^ and cd73^-/-^ microglia was significantly smaller than that covered by wild-type microglia, namely, 516 ± 58 μm^2^ and 210 ± 23 μm^2^, respectively (Fig 8B). This spontaneous motility in wild-type, cd39^-/-^ and cd73^-/-^ microglia was completely abrogated in the presence of ticagrelor (5 μM) (Fig 8B).

### cd73^-/-^ and cd39^-/-^ cultured microglia show a less complex morphology compared to wild-type microglia

Cultured neonatal microglia are not ramified but can acquire different morphological phenotypes when cultivated under serum-free conditions and in the absence of an astrocyte feeder layer. To characterize the morphological heterogeneity of cultured microglia, we determined the area and the circularity value of individual cells in a population of wild-type, cd39^-/-^ and cd73^-/-^ microglia (see materials and methods) (Fig 8C). Circularity is a numerical value representing cell shape; cell circularity can range from 1 (perfect circle) to lower values indicating a cell with protrusions or processes. Wild-type microglia (N = 2590) varied in cell circularity from 0.06 to 0.98 (median 0.37) (Fig 8D) and in cell area from 200 μm^2^ to 2000 μm^2^ (median 734 μm^2^) (Fig 8E). cd73^-/-^ (N = 2490) and cd39^-/-^ (N = 1100) microglia had larger circularity values: 0.63 and 0.54, respectively (Fig 8D), but the cell area was on average 733 μm^2^ and 860 μm^2^, respectively and did not significantly (p>0.5) differ from values of wild-type microglia (Fig 8E). Histograms show that in the population of wild-type microglia, the highest number of cells was found with circularity values between 0.2 and 0.5, but knockout microglial cells were evenly distributed through the entire range of circularities, supporting the finding *in vivo* that cd39^-/-^ and cd73^-/-^ microglia exhibit a less complex morphology.

### Adenosine promotes a ramified microglial phenotype *in vitro*

**Fig 9:**
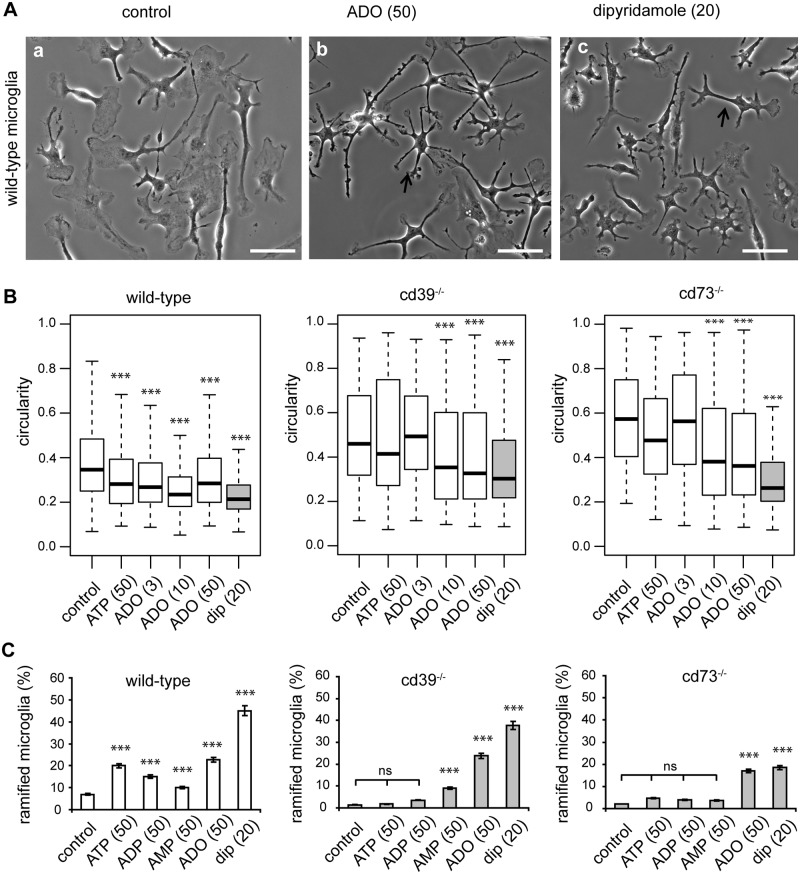
Adenosine is required for the transformation of microglia into a ramified phenotype *in vitro*. Microglia were cultivated for 24 h in serum-free medium alone or in the presence of ATP, adenosine or dipyridamole. (A) Phase-contrast images of wild-type microglia in control conditions (left image), in the presence of adenosine (ADO, 50 μM, middle image), or dipyridamole (20 μM, right image) are shown. Arrows mark ramified microglial cells with a circularity < 0.3. Scale bar is 20 μm. (B) Box plots show the distribution of cell circularities of microglia from wild-type (left), cd39^-/-^ (middle) and cd73^-/-^ mice (right) as well as knockout microglia in control solution or after addition of ATP (50 μM), adenosine (ADO, 3 μM, 10 μM, 50 μM) or dipyridamole (dip, 20 μM). Data show circularities of at least 200 cells per one experiment out of 3 independent experiments. (C) We classified cells with a circularity < 0.3 as ramified and plotted the proportion of ramified cells in a population of wild-type (left, white bars), cd39^-/-^ (middle, grey bars) and cd73^-/-^ (right, grey bars) microglia. Cells were cultivated in medium alone (control) or in the presence of ATP (50 μM), ADP (50 μM) and AMP (50 μM), adenosine (ADO, 50 μM) or dipyridamole (dip, 20 μM). Significant differences were tested by comparison of multiple proportions. *** denotes p < 0.001 in comparison to control.

Next, we tested the impact of extracellular nucleotides and adenosine on the morphological complexity of cultured microglia. When wild-type microglia were cultivated for 24 h in the presence of ATP, adenosine or dipyridamole, cells gained a significantly more ramified phenotype. In untreated controls, the circularity value was 0.35; addition of ATP (50 μM) reduced it to 0.28; adenosine (3, 10, 50 μM) reduced it to 0.27, 0.23, 0.28, respectively; and dipyridamole (20 μM) reduced it to 0.21 (N = 250, each condition) (Fig 9A and 9B). In the knockout microglia cultures, constitutive circularity values were higher compared to wild-type controls, namely, 0.46 and 0.57 for cd39^-/-^ and cd73^-/-^, respectively. Addition of 50 μM (Fig 9B) or 100 μM (not shown) ATP or 3 μM adenosine did not induce a significant (p>0.5) change in cell circularity, but 10 μM and 50 μM of adenosine and dipyridamole significantly (p<0.01) decreased cell circularity to 0.35, 0.33 and 0.30 in cd39^-/-^ microglia or to 0.38, 0.36 and 0.26 in cd73^-/-^ microglia, respectively (N = 250, each condition) (Fig 9B). Cultivation of wild-type microglia for an additional 4 days further decreased cell circularity to 0.21 in control and to 0.12, 0.10 and 0.09 in the presence of ATP (50 μM), adenosine (50 μM) and dipyridamole, respectively. Interestingly, cultivation under serum-free conditions and in the absence of astrocytes as a feeder layer supported the survival of wild-type but not of knockout microglia; 80% and 40% of wild-type microglia survived by day 5 and day 10, respectively, whereas 80% of cd39^-/-^ and cd73^-/-^ microglia died by day 5.

In another approach, we defined a circularity threshold of 0.3 to subdivide microglia into two populations that were either more or less ramified (Fig 9A). Cells with a circularity value less than 0.3 typically had small soma and up to 6 thin processes that extended away from the cell soma; the average process length was 25 ± 15 μm and 15 ± 10 μm in wild-type and knockout microglia, respectively. In control conditions, 7% of wild-type cells, but only 1% of knockout cells were classified as more ramified. The population of more ramified wild-type microglia was significantly increased in the presence of ATP, ADP, AMP, adenosine and dipyridamole (Fig 9C). In cd39^-/-^ microglia, only AMP, adenosine and dipyridamole but not ATP or ADP significantly increased the population of more ramified microglia, while in cd73^-/-^ microglia, only adenosine and dipyridamole led to a significant increase in the more ramified population (Fig 9C). Time-lapse video microscopy of such cultured ramified cells revealed that process-like protrusions were long-lasting (persisted for at least 5 h or longer) and were not retracted, but the edges of such protrusions contained a dynamic lamellipodium-like structure that constantly underwent retraction and protrusion cycles. Notably, regardless of the genotype, the presence of ticagrelor (5 μM) in the cell culture medium prevented a transformation of microglia into the more ramified phenotype but caused edges and protrusions to retract (Fig 9A). These results indicate that the ramified phenotype of cultured microglia is promoted by extracellular adenosine, and its availability is regulated by CD39, CD73 and equilibrative nucleoside transporter 1 (ENT1).

## Discussion

Microglia enter the brain in an amoeboid form during development and transform into a ramified phenotype. In the healthy brain, all microglial cells exhibit this highly branched morphological phenotype. The factors that trigger this morphological transformation are not well studied as of yet. Rio-Hortega (1919), in the first papers describing microglia, considered the amoeboid form as the default morphological phenotype of microglia. He argued that the brain microenvironment, by, to him, unknown factors, transforms microglia into the ramified phenotype [40]. Several studies in cell culture indicate that astrocytes influence the process of ramification of microglia and transforming growth factor-beta (TGF-β), macrophage colony-stimulating factor and granulocyte/macrophage colony-stimulating factor are relevant stimuli released by astrocytes [41, 42]. ATP, adenosine, Vitamin E, IL-34 and chemokine fractalkine were other factors which were proposed to induce microglia ramification *in vitro* [43–47]. Inhibition of endoplasmic reticulum Ca2-ATPase by thapsigargin, down-regulation of complement receptor 3 and major histocompatibility complex I and II antigen-like immunoreactivity accompanied ramification of microglia *in vitro* [48, 49]. In the postnatal mouse brain microglia are initially amoeboid but as brain development proceed they gradually undergo a transition into a ramified state and a transcriptional factor Runx1 was found to be expressed in postnatal amoeboid microglia but it was down regulated in adult ramified microglia [50, 51]. This ramification process is recapitulated in reverse during microglia activation in response to brain injury or disease [52–54]. Retraction of microglial processes during injury was coincide with an up regulation of adenosine receptors 2A and down regulation of P2Y(12) receptors [15, 19]. In adult TGF- β1^-/-^ and Sall1^-/-^ mice microglia had more rounded appearance [55, 56]. Superramified morphology with numerous branched processes was demonstrated in remaining microglial cells after microglia depletion [57] and in microglial cells on contralateral binocular visual cortex in the model of monocular deprivation [58]. In the healthy adult brain, microglial cells constantly survey the environment by moving their processes and thus are the most highly motile elements in the central nervous system [5, 6].

In the present study, we addressed the question of which factors control the ramified microglial phenotype. Braun et al. (2000) have previously demonstrated that microglial cells in cd39-deficient animals have a less complex morphology [26]. We have focused on extracellular adenosine as a potential regulator of the formation and ramification microglial processes under normal, non-pathological conditions. Here, we have analyzed microglial morphology in the brain, in the acute brain slice and in cell culture in a quantitative fashion. We demonstrated that CD39-deficient microglia have a less arborized phenotype due to a decrease in the cumulative length of processes and a reduction in the number of branch points and Sholl sphere intersections. In CD73-deficient microglia, we found a similar morphological alteration. CD39 controls the degradation of ATP or ADP into AMP, while CD73 generates adenosine from AMP [22, 25]. Thus adenosine generated in the microglia microenvironment by the CD39/CD73 enzyme system is directly involved in a ramification of microglia processes.

Our data indicate that co-stimulation of P2 and P1 receptors is required to generate the complex ramified morphology of microglia and that CD39 and CD73 activity is required for generating adenosine. ATP does not seem to be the limiting ligand since it is released from many cells by mechanisms as discussed below [59]. To study the dynamics of process extension, we used both acute brain slices and cultured microglial cells as experimental paradigms. In wild-type animals, ATP or ADP leads to process elongation in microglia, while in cd39-deficient animals, an additional stimulation by adenosine is required. Thus, microglial process extension occurs by combined stimulation of ATP and adenosine. We also found a less complex morphology of cultured neonatal microglial cells from cd39- and cd73-deficient animals, and similar to the results observed in acute brain slices, ATP and ADP were able to increase morphological complexity of microglia in wild-type animals but not in cd39^-/-^ and cd73^-/-^ animals. More evidence supporting our hypothesis comes from our observations that the non-hydrolysable form of ADP, 2meS-ADP, did not trigger process extension in acute brain slices of wild-type mice and that apyrase, which degrades ATP/ADP to AMP, induced microglial process elongation in cd39^-/-^ mice. Thus, ATP/ADP breakdown is required for ramification of microglial cells under normal conditions.

We found that P2Y12 receptors are required for the ramification of microglia by using the specific inhibitor ticagrelor [60]. We also used dipyridamole, an inhibitor of equilibrative nucleoside transporter 1 (ENT1), as a tool to elevate adenosine in the extracellular environment. Dipyridamole has indeed been shown to lead to an elevation of extracellular adenosine concentration and thus enhances the activation of adenosine receptors [33, 61, 62]. Dipyridamole augmented anti-inflammatory, anti-oxidant and vasodilatory effects of adenosine in different experimental models [21, 63, 64] including the reduction of microglia activation in the mouse model of multiple sclerosis [65]. All these data indicate that co-stimulation of purinergic and adenosine receptors promotes a ramified microglial phenotype and that combined CD39 and CD73 activity regulates the adenosine supply.

While the ramified phenotype per se has not been widely studied, the mechanisms underlying cell migration and chemotaxis have been more extensively analyzed [66, 67]. Both types of cellular responses depend on purinergic signaling. Microglial chemotaxis depends on purinergic signaling via P2Y receptors as first observed by Honda et al. (2001) [12]. We previously observed that migration of microglia was affected by CD39 deficiency and that in these cd39 knockout microglia, co-stimulation of P2 and P1 receptors was required for proper migration [27]. Similarly, co-stimulation of P2Y2 receptors and A3 adenosine receptors is required for chemotaxis in neutrophils [28, 68, 69]. Thus both, chemotaxis and chemotactic process movement requires dual stimulation by the P2Y and adenosine receptor system as recently reviewed by Koizumi et al. (2013) [19].

The motility of processes strongly depends on P2Y12 receptor activity as shown by the impairment of process motility in P2Y12-deficient animals [7]. Dissing-Olesen et al. (2014) reported that ATP release secondary to NMDA receptor activation triggers the growth of microglial processes [11]. Eyo et al. (2014) similarly demonstrated that release of ATP triggered by activation of NMDA receptors leads to process extension of microglia [70].

In the cd39-deficient animals, a potentially higher level of ATP might additionally affect adenosine generation by CD73 activity since ATP and ADP, as well as their non-hydrolysable analogues, are competitive inhibitors of ecto-5’-nucleotidase/CD73 with inhibition constants in the low micromolar range [71]. ATP and ADP, such as AMP, can bind to the active sites of CD73, but they cannot be hydrolyzed to adenosine. Interesting, ATPγS, a metabolically stable analog of ATP, was unable to substitute for ATP in the stimulation of microglia process elongation *in vivo* [5].

Under pathological conditions, adenosine A2 receptors are upregulated, become important for the control of process activity, and mediate microglial process retraction [15]. Thus, under pathological conditions, the receptor repertoire expressed by microglia can change, and these changes trigger an amoeboid phenotype.

ATP can be released into the extracellular space virtually from every cell [59]. Thus, multiple ATP release sites exist in the normal brain [72]. ATP acts as a neurotransmitter and is released during synaptic transmission [73]. Astrocytes release ATP and thereby generate Ca^2+^ waves as a propagating signal [74]. We have previously demonstrated that microglial cells sense the activity of the astrocyte calcium waves via activation of purinergic receptors [75]. Endogenously released ATP triggered by activation of purinergic receptors is involved in long-range microglia chemotaxis [76]. The mechanisms of ATP release are heavily debated and can include release via vesicles, hemichannels or even P2X7 receptors [77]. Thus, microglia are indeed constantly exposed to ATP, and our results indicate that this chronic exposure promotes their ramified phenotype. Duration and frequency of the interaction of microglial processes with synapses is also modulated by neuronal activity [9, 10]. The process of brain slicing can be considered as a pathologic event; we showed that in acute brain slices, the process length and complexity of microglia are reduced in wild-type mice and are even further reduced in cd39^-/-^ mice.

We found that deletion of cd39, cd73 or both of these genes caused a 20% reduction in microglia density in the cortex and strongly limited the survival of knockout microglia *in vitro*. Additionally, dipyridamole inhibited microglial survival. Thus, CD39 and CD73 enzymes together with ENT1 can regulate the fate of extracellular nucleotides and adenosine in the microglial microenvironment, thereby modulating microglia process motility and survival. In conclusion, these findings demonstrate that under normal physiological conditions, extracellular adenosine that is generated by CD39/CD73 nucleotidases is required for ramification of microglial processes *in vitro* and *in vivo*.

## Supporting information

S1 Fig Constitutive deletion of CD39 and/or CD73 attenuates spatial distribution of microglial process. An example of a symmetric (A) and non-symmetric (B) microglia cell in somatosensory cortex of a wild-type mouse; pink dots represent cell soma, green dots show process terminals (terminal points), quadrants correspond to four directions (dorsal _left_, dorsal _right_, ventral _left_ and ventral _right_) on coronal brain slice; these directions are shown in (D) on a confocal fluorescence image of an iba-1/Alexa fluo-488 labelled coronal brain slice of a wild-type mouse, white arrows show the direction; scale bar 1000 μm. The percentage of process terminals is shown for each quadrant; in a symmetric cell (A) every quadrant contains 25 ± 10% but in non-symmetric cell (B) at least one quadrant contains less than 15% or more than 35% of the cumulative number of process terminals. Quantification of the distribution of process terminals around cell soma demonstrates that in adult (P56) cd39^-/-^, cd73^-/-^, and double knockout mice individual microglia cells are significantly less symmetric in comparison to microglia from wild-type mice (C). Individual microglial cells from wild-type (grey, N = 46), cd39^-/-^ (pink, N = 46), cd73^-/-^ (green, N = 70) and double knockout (cd39^-/-^/cd73^-/-^) (blue, N = 128) mice were analyzed using Imaris 6.7.4 (see Figs 1 and 2); N represents the number of individual cells. Significant difference was tested by one-way ANOVA, *** denotes p < 0.001.
(TIF)
