# Supplementary figures and images for: The adenosine generating enzymes CD39/CD73 control microglial processes ramification in the mouse brain
Source: PLoS One. 2017 Apr 4;12(4):e0175012. doi: 10.1371/journal.pone.0175012 (PMC5380357; doi:10.1371/journal.pone.0175012)

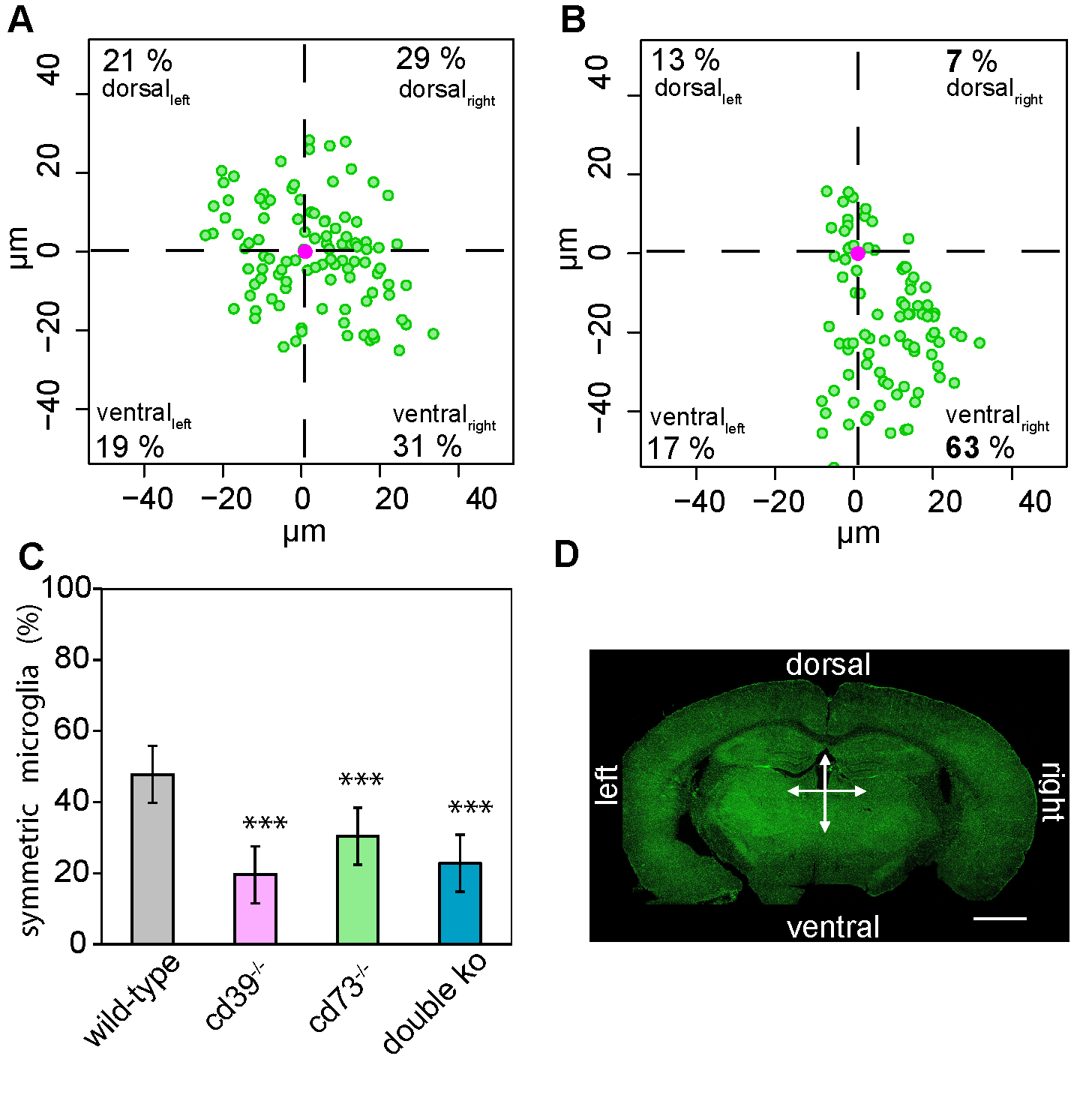

Supplement: S1 Fig — An example of a symmetric (A) and non-symmetric (B) microglia cell in somatosensory cortex of a wild-type mouse; pink dots represent cell soma, green dots show process terminals (terminal points), quadrants correspond to four directions (dorsal left, dorsal right, ventral left and ventral right) on coronal brain slice; these directions are shown in (D) on a confocal fluorescence image of an iba-1/Alexa fluo-488 labelled coronal brain slice of a wild-type mouse, white arrows show the direction; scale bar 1000 μm. The percentage of process terminals is shown for each quadrant; in a symmetric cell (A) every quadrant contains 25 ± 10% but in non-symmetric cell (B) at least one quadrant contains less than 15% or more than 35% of the cumulative number of process terminals. Quantification of the distribution of process terminals around cell soma demonstrates that in adult (P56) cd39-/-, cd73-/-, and double knockout mice individual microglia cells are significantly less symmetric in comparison to microglia from wild-type mice (C). Individual microglial cells from wild-type (grey, N = 46), cd39-/- (pink, N = 46), cd73-/- (green, N = 70) and double knockout (cd39-/-/cd73-/-) (blue, N = 128) mice were analyzed using Imaris 6.7.4 (see Figs 1 and 2); N represents the number of individual cells. Significant difference was tested by one-way ANOVA, *** denotes p < 0.001. (TIF) [file pone.0175012.s001.tif]
